# Experiential Faculty Development to Increase the Number of Entrustable Professional Activity Assessments

**DOI:** 10.1111/tct.70006

**Published:** 2024-12-25

**Authors:** Michael Buyck, Pierre Desaulniers, Christophe Chénier, Ahmed Moussa

**Affiliations:** ^1^ Faculty of Medicine University of Geneva Geneva Switzerland; ^2^ Pediatric Emergency Department Geneva University Hospitals Geneva Switzerland; ^3^ Centre for Applied Health Sciences Education (CPASS), Faculty of Medicine Université de Montréal Montreal Quebec Canada; ^4^ Emergency Medicine Centre Hospitalier de l'Université de Montréal Montreal Quebec Canada; ^5^ Faculty of Education Science Université de Montréal Montreal Quebec Canada; ^6^ Departement of Pediatrics, Division of Neonatology, Centre Universitaire Hospitalier Sainte‐Justine Université de Montréal Montréal Quebec Canada; ^7^ CHU Sainte‐Justine Research Centre Université de Montréal Montréal Quebec Canada

**Keywords:** emergency medicine, entrustable professional activities, faculty development

## Abstract

**Background:**

Emergency medicine (EM) residents must complete both adult and paediatric entrustable professional activities (EPAs). During their paediatric emergency medicine rotation at a university paediatric hospital, EM residents struggled to receive EPA assessments because preceptors had not yet been trained due to the stepwise implementation of EPAs. This study aimed to evaluate the impact of a workshop on behaviour change by measuring the number of EPA assessments.

**Methods:**

A comparative pretraining and posttraining study involving 27 invited faculty members was conducted to assess the impact of a faculty development programme. The training was delivered via videoconference with experiential learning techniques to practise every aspect of the supervision of an EPA, including selecting the appropriate EPA according to mirroring real‐world situations, giving feedback, evaluating autonomy and recording the EPA in the resident's logbook.

**Results/Findings:**

In total, 20 out of 27 eligible faculty members (74%) agreed to participate in the study. Their main challenges reported were a lack of trainee initiative, preceptor training and competence in supervising EPAs. Over the 12‐month analysis period, the enrolled faculty assessed 125 EPAs for 38 EM residents, including 52 pre‐intervention EPAs and 73 post‐intervention EPAs. Calculation of data points above the median showed a 1‐point difference in the EPAs assessments to resident ratio between the pre‐ and post‐intervention periods (3/7 vs. 4/7).

**Conclusion:**

Our findings suggest that faculty training using multiple educational strategies may enable EM residents to receive more EPA assessments during their paediatric emergency medicine rotation.

## 1 Background

In line with many countries, emergency medicine (EM) physicians in Canada are trained to care for both adult and paediatric patients [1]. This specialised training is achieved through mandatory rotations in paediatric emergency departments and the assessment of context‐specific paediatric entrustable professional activities (EPAs). EPAs are tasks or responsibilities of increasing complexity and scope as residency training progresses [2]. Indeed, at the end of training, EPAs reflect abilities approaching those of a practising physician. Although some EPAs can be completed by residents independently, most require observation and assessment by a faculty member. Following feedback, EPAs are aggregated in a digital platform.

Residents face challenges in obtaining assessments for their EPAs, according to several studies, particularly during ectopic rotations, that is, rotations held outside their regular training environment [3, 4]. EPA assessments necessitate direct observation and evaluation by unfamiliar supervisors in a new setting [5, 6]. In some settings, particularly in paediatric emergency departments, supervisors have not received adequate training in assessing EPAs. This gap arose due to the phased implementation of EPAs in university hospital training settings where the paediatric programme was scheduled to follow the EM programme [2]. However, this timeline was disrupted by the unexpected impact of the COVID‐19 pandemic, resulting in a lack of preparedness for EPA assessments in paediatric departments hosting EM residents. Consequently, supervisors often failed to assess the recommended one EPA per shift, as suggested by EM programmes. A needs assessment conducted prior to this study identified two major barriers to effective EPA assessment: a lack of understanding of the EPA concept and insufficient guidance on how to conduct EPA assessments (unpublished data). As a result, the absence of proper supervisor training significantly limited the number of EPA assessments completed for EM residents.

“In some settings, particularly in paediatric emergency departments, supervisors have not received adequate training in assessing EPAs.”

To ensure successful implementation of EPAs within residency programmes, faculty development is essential to inform and equip supervisors with effective pedagogical strategies [7]. This supervisor‐focused training facilitates acquisition of direct observation techniques, feedback and coaching methods, as well as the integration of the notion of entrustment [8, 9]. The Royal College of Physicians and Surgeons of Canada provides online resources [10, 11], and universities offer training [12] for physicians; however, there has been a paucity of research conducted to evaluate the effectiveness of such training in changing supervisors' behaviour. Therefore, we designed a faculty development workshop and aimed to confirm its positive effect on the number of EPA assessments by supervisors.

“There has been a paucity of research conducted to evaluate the effectiveness of such training in changing supervisors' behaviour.”

## 2 Methods

### 2.1 Study Design

We conducted a comparative pretraining/posttraining study from September 2020 to August 2021. The aim of the study was to measure the number of EPAs assessed by supervisors following a faculty development workshop. This objective aligns with a third‐level outcome as defined by Kirkpatrick's model. This framework, assessing the impact of training, comprises four levels of outcome: reaction, learning, behaviour and results. The first level assesses participants' immediate reactions to the training, such as satisfaction and engagement. The second level measures the knowledge or skills acquired. The third level examines changes in behaviour and the application of new skills in the workplace. The fourth level evaluates the overall impact on organisational performance and outcomes.

### 2.2 Settings

The study was conducted at the *Centre Hospitalier Universitaire de Sainte‐Justine* (CHUSJ), a paediatric trauma centre and maternity hospital in Montreal, Canada, which sees more than 90,000 paediatric emergency department visits annually. Around 40 EM residents undergo training in the emergency department every year.

There are two programmes that train emergency physicians in Canada: the 5‐year Residency Programme in Emergency Medicine (RCPSC‐EM) and the 12‐month Certification in Family Medicine with Added Competency in Emergency Medicine (CCFP‐EM). Residency programmes comprise 13 four‐week periods per year (P1 to P13). Most RCPSC‐EM and CCFP‐EM residency programmes in Quebec require several paediatric emergency rotations at CHUSJ. During these rotations, residents manage patients over 12 to 16 eight‐hour shifts. Each consultation is under the supervision of a designated physician for the entire shift. The plan of care is established by the supervisor after the patient's assessment and may include additional tests, treatments and consultations with specialists. Residents, except those working in resuscitation rooms or doing procedures, receive indirect supervision, which means that the supervisor does not observe the resident taking a history or performing a physical examination.

Since 2017, the RCPSC‐EM programme has transitioned to competency‐based training using the competency‐by‐design model [2]. This training framework is a hybrid of competency‐based and time‐based training, meaning that the typical training timelines still apply, but residents have the opportunity to progress faster (or slower) depending on their measured competency. As part of the assessment programme, residents must validate a specified number of EPAs with emergency paediatric patients in order to meet their requirements. Since 2009, the CCFP‐EM programme has used a competency‐based strategy based on the College of Family Physicians of Canada's Triple C model [13]. The programme includes EPAs created in Quebec, with 4 of 11 being specific to paediatric patients. Both programmes require their residents to complete at least one paediatric emergency rotation at the CHUSJ to fulfil the requirements of the paediatric EPAs. Both programmes suggest that at least one EPA should be assessed per shift.

### 2.3 The Participants

Participants in this study were physicians who were board certified in paediatric EM (PEM) by the RCPSC and were supervisors of residents at CHUSJ. The workshop was supported by the department and scheduled as part of the department's weekly academic day for all faculty. Two dates were offered in February 2021 to allow as many doctors as possible to attend. In addition, an email invitation was sent to each physician. Physicians were also allowed to participate in the training without being enrolled in the study. There were no exclusion criteria. Informed consent was obtained from each participant before demographic information was collected. No compensation was given.

### 2.4 Training Session for Participants

The training session consisted of a 2‐h workshop led by M.B. and P.D. MB, a paediatric emergency medicine fellow at CHUSJ and a student in the Master's programme in medical education at the *Université de Montréal*, and P.D., the director of the RCPSC‐EM programme and a professor with extensive experience in competency‐based education reforms in Quebec, conducted the workshop. Due to health restrictions during the COVID‐19 pandemic, the workshop was delivered exclusively online via the Zoom platform (San Jose, USA). Physicians who were unable to attend the live online workshop were given the option to view a recorded version, ensuring they had access to the same training materials and information as those who participated in real time. The workshop was divided into four main sections (detailed descriptions of each section are provided in Table 1):

1. Introduction to EPAs: overview of the concept of EPA, with a focus on those specific to PEM for RCPSC‐EM and CCFP‐EM residents [14], using a rotation card created to guide EPA selection [15] (Appendix S1).
2. Supervision practice: hands‐on practice in supervising an EPA through five clinical scenarios, including EPA selection, observation, feedback and evaluation.
3. Electronic system overview: introduction to the electronic system for recording EPA assessments.
4. Feedback practice: exercises and discussion on providing effective feedback, with tips and tricks shared by instructors [16].

We utilised faculty development best practices like employing multiple pedagogical approaches, utilising active and experiential learning techniques such as the use of an audience response system (Poll Everywhere, Poll Everywhere Inc.) and providing opportunities for feedback through Q&A sessions [17, 18, 19].

**TABLE 1:** Detailed overview of workshop sections.

Section	Description	Duration
Introduction to EPAs	‐ Introduction to the concept of EPAs specific to paediatric emergency medicine for RCPSC‐EM and CCFP‐EM residents.	20 min
	‐ Use of a specific rotation card to guide EPA selection.	
	‐ EPAs covered: F1, F2, F4, C1, C2, C3, C4, C5, C6, C9, C11 (specific to the paediatric setting).	
Supervision Practice	‐ Practice in five clinical scenarios involving EPA selection, observation, feedback and evaluation.	60 min
	‐ Scenarios included settings like performing local finger anaesthesia, with faculty collaborating on EPA selection and providing feedback.	
	‐ Use of prerecorded video or audio illustrations.	
Electronic System	‐ Presentation and demonstration of the electronic system used to record EPA assessments.	10 min
Feedback Practice	‐ Exercises, tips and tricks for giving effective feedback in various situations.	30 min
	‐ Faculty shared their experiences and challenges, with instructors providing practical advice.	

Abbreviations: CCFP‐EM, Certification in Family Medicine with Added Competency in Emergency Medicine; EPA, entrustable professional activity; RCPSC‐EM, Royal College of Physicians and Surgeons of Canada–Residency Programme in Emergency Medicine.

### 2.5 Data Collection

At the beginning of the training, demographic information was collected using an online form (Microsoft Forms, Microsoft Corporation). The questionnaire asked about general demographic information, teacher characteristics and experiences or training related to EPAs.

Data on the number of EPAs per period were retrieved from the RCPSC‐EM and CCFP‐EM residency programme databases over a 12‐month analysis period, which spanned from 24 August 2020 to 29 August 2021. This period included six‐and‐a‐half training periods before the intervention (from Period 3 to September 2020) and six‐and‐a‐half periods after the intervention (up to Period 2 in August 2021). Period 9 was split in two, as the intervention was implemented midway through the period. For consistency and ease of analysis, the 12‐month timeframe was used.

### 2.6 Analysis

Descriptive statistics for the number of EPA assessments were calculated for each phase (pretraining and posttraining) and were plotted as a box‐and‐whisker plot for the two phases. To account for variations in the number of residents in each rotation, we calculated the ratio of recorded EPAs to the number of residents present during each period, rather than using the absolute number of EPAs performed in each training period. The median was calculated for each phase, and the percentage of points exceeding this median was compared for each phase [20]. Analyses were performed using R (R Foundation for Statistical Computing v4).

### 2.7 Ethics Statement

The study was approved by the ethics committee of CHUSJ in Montréal, Canada.

## 3 Findings

Out of the 27 eligible PEM physicians, 20 (74%) consented to participate in the study. Five physicians did not respond, and two explicitly declined to participate. Among those who consented, 18 (66%) attended the workshop while the remaining 2 participants, who were unable to attend, were provided with access to the recorded session. Most of our workshop participants were women (12 out of 18), supervising more than 12 residents per 4‐week rotation period and trained in feedback but not in EPA assessments. Regarding the participants' pedagogical motivations, they stated that they prefer to work with trainees and/or residents and that they quickly adopt new educational strategies (Table 2). Among the challenges in assessing EPAs, lack of preceptor training and competence in supervising EPAs were the most frequent. Other challenges included lack of trainee initiative, and lack of time during shifts (Table 3).

“Among the challenges in assessing EPAs, lack of preceptor training and competence in supervising EPAs were the most frequent.”

**TABLE 2:** Participant demographics, including training and experience.

Demographics	Participants (*n* = 18)
Female	12 (77%)
Age (years)	
30 to 39	7 (39%)
40 to 49	3 (17%
50 to 59	8 (44%)
Paediatric emergency experience (years)	
0 to 5	6 (33%)
6 to10	1 (6%)
11 to 15	2 (11%)
16 to 20	4 (22%)
21 to 25	5 (28%)
How many trainees and/or residents do you supervise during each 4‐week training period?	
7 to 9	4 (22%)
10 to 12	2 (11%)
More than 12	12 (67%)
When working in the paediatric emergency department, if given a choice, what type of supervision would you prefer?	
Always without trainees and/or residents	0 (0%)
Usually without trainees and/or residents	0 (0%)
No preference	3 (17%)
Usually with trainees and/or residents	9 (50%)
Always with trainees and/or residents	6 (33%)
Have received training in feedback	10 (66%)
Have received training in EPA	3 (17%)
How do you react to a new teaching approach?	
I am waiting to see how it works for others.	2 (11%)
I think about it.	3 (17%)
I think about it, but I adopt it quickly.	9 (50%)
I adopt it right away.	4 (22%)
Experience in using EPAs (months)	
3 or less	4 (22%)
3 to 6	4 (22%)
6 to 12	2 (11%)
12 to 24	6 (33%)
More than 24	1 (6%)
Never	1 (6%)

Abbreviation: EPA, entrustable professional activity.

**TABLE 3:** Experiences and perceived representations of entrustable professional activities (EPA) prior to intervention.

Opinions regarding EPAs	Participants (*n* = 18)
What is an EPA for you?	
A form of feedback	17 (94%)
A teaching method	10 (56%)
An evaluation method	17 (94%)
An administrative obligation	10 (56%)
An opportunity for direct observation	16 (89%)
What are the difficulties associated with EPAs?	
I have no situation that merits an EPA	1 (6%)
I have not had any training	11 (66%)
I do not have the time to do an EPA	11 (66%)
I'm not familiar with residents' EPAs	12 (67%)
I do not know how to do an EPA	6 (33%)
Residents do not ask me for an EPA	16 (89%)
I'm not on the list of EPA supervisors	1 (6%)

**FIGURE 1:**
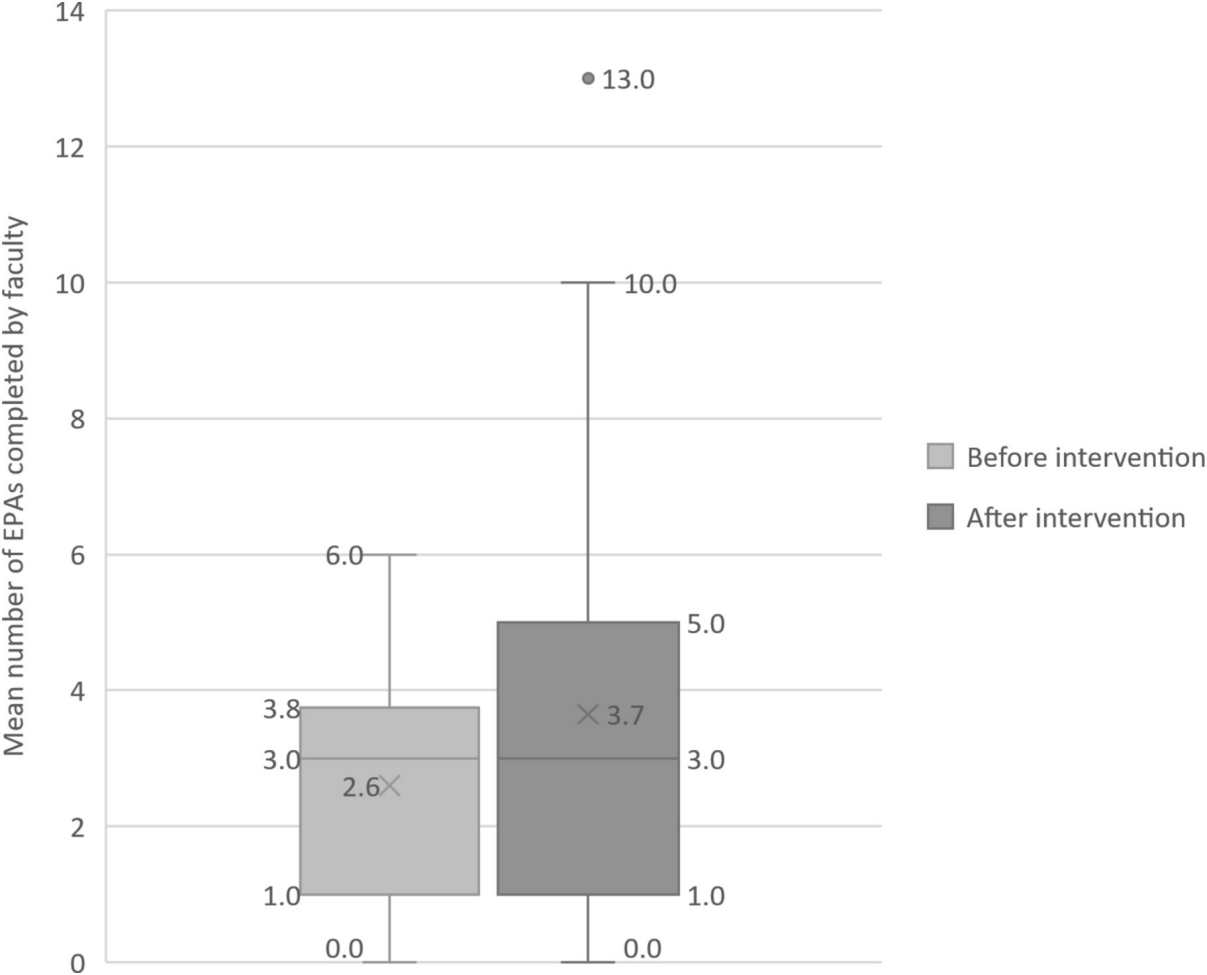
EPAs completed by participants before and after the intervention. EPA, entrustable professional activity.

The 12‐month analysis period was from 24 August 2020 to 29 August 2021 (Table 4). During these 12 months, 141 EPAs were completed for 38 EM residents (6 RCPSC‐EM residents, 32 CCFP‐EM residents) by 29 different supervisors. Of the 141 EPAs, 125 (89%) were completed by 20 participants (the remaining 16 EPAs were completed by nonparticipating or ineligible physicians), including 52 pre‐intervention EPAs (mean 2.6 EPAs per supervisor [CI 95% 2.5–2.7]) and 73 post‐intervention EPAs (mean 3.7 EPAs per supervisor [CI 95% 2.1–5.1]), as shown in Figure 1. The median number of EPAs per supervisor remained unchanged between the two periods (three EPAs per supervisor).

**TABLE 4:** Number of EPAs, number of residents and EPA to resident ratio over the study period.

Rotation period	Date	Number of EPAs	Number of EM residents	EPA to resident ratio
P03 2020	24 August to 20 September 2020	0	0	0.0
P04 2020	21 September to 18 October 2020	1	2	0.5
P05 2020	19 October to 15 November 2020	15	3	5.0
P06 2020	16 November to 13 December 2020	10	2	5.0
P07 2020	14 December to 10 January 2021	23	3	7.7
P08 2020	11 January to 7 February 2021	6	3	2.0
P09a 2020	8 February to 22 February 2021	4	3	1.3
P09b 2020	23 February to 7 March 2021	6	3	2.0
P10 2020	8 March to 4 April 2021	20	6	3.3
P11 2020	5 April to 2 May 2021	11	6	1.8
P12 2020	3 May to 30 May 2021	7	2	3.5
P13 2020	31 May to 30 June 2021	1	3	0.3
P01 2021	1 July to 1 August 2021	15	2	7.5
P02 2021	2 August to 29 August 2021	22	3	7.3

*Note:* EM residents include both programmes; Period 2020 P09 is divided into two parts (a and b) because the workshop was given in the middle of this period.

Abbreviations: EPA, entrustable professional activity; EM, emergency medicine.

**FIGURE 2:**
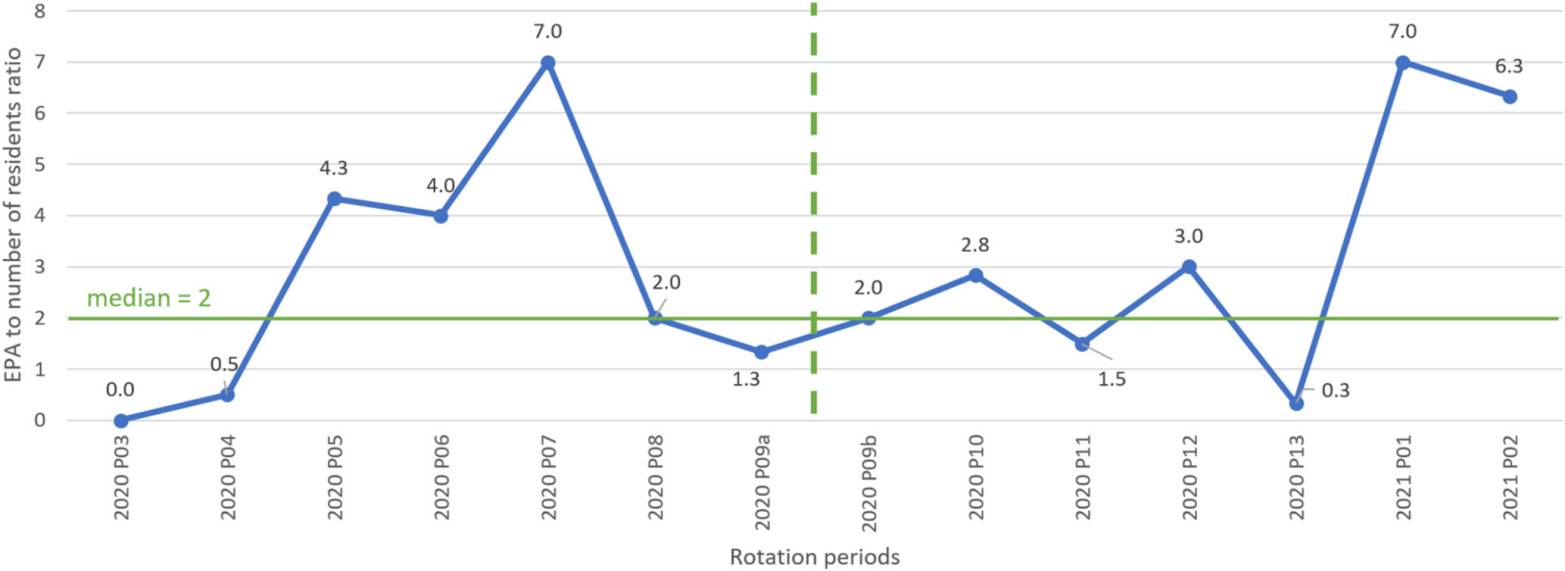
Representation of data points above the median during the pre‐ and post‐intervention periods (period 2020 P09 is divided into two parts (a and b) because the workshop took place in the middle of this period). EPA, entrustable professional activity.

The number of residents per period ranged from 0 to 6. The time series in Figure 2 shows the ratio of the number of EPA assessments to the number of residents present during each period. Calculation of data points above the median showed a difference of 1 between the pre‐intervention and post‐intervention periods (3/7 vs. 4/7, respectively).

A supervisor‐by‐supervisor calculation of data points exceeding the median was not possible due to the small number of EPAs completed by each supervisor. However, it is worth noting that nine participants completed more EPAs after the workshop than before, with an increase ranging from 1.1× to 11× between the two periods.

## 4 Discussion

In this study, we evaluated the effectiveness of a faculty development workshop designed to enhance PEM physicians' ability to assess EPAs for EM residents during their rotations in the paediatric emergency department. The intervention led to a marked increase in the number of EPA assessments, which is a promising indicator of more active supervision and improved feedback processes. Given that EM residents must develop competencies in managing paediatric emergencies and considering that this ectopic rotation is one of their few opportunities to practice under expert supervision in paediatric care, the increase in EPA assessments is particularly significant.

Our findings demonstrate that the experiential faculty development intervention contributed to an increase in the number of EPA assessments conducted by PEM physicians. This outcome suggests a positive behavioural change among faculty, addressing a critical need identified in our earlier needs assessment. This contrasts with the findings of Sood et al. [21] and Rivkin et al. [8], which respectively reported improvements in appreciation and learning rather than direct behavioural changes in assessment practices. The two studies, which were conducted as a 90‐min workshop and a short course, respectively, comprised an introduction to CBME and EPA assessment, small‐group practice on a written vignette and a concluding session.

“Our findings demonstrate that the experiential faculty development intervention contributed to an increase in the number of EPA assessments conducted by PEM physicians.”

Our intervention resulted in a modification of faculty behaviour as it comprises a range of faculty development components suggested by Stefan et al. [6]. First and foremost, we promoted *intentional community building* within the department: The faculty showed immediate enthusiasm for mentoring EM residents who are required to complete EPAs because they come back for further rotations in the department and will be practicing paediatric medicine in the future. Similar to Rivkin's research, our training efforts were concentrated on a *selection of relevant content*, particularly the presentation and application of EPAs specific to the paediatric emergency setting. Furthermore, we placed emphasis on *clarifying* the EPAs, as we know that their terminology can be a barrier to their completion [22]. Additionally, developing a rotation card that summarises the EPAs specific to the rotation contributes to this clarification and helps faculty select the most appropriate EPA for the clinical situation being supervised [15] (see Appendix S1).

*Experiential learning*, *feedback opportunities* and *reflection* were central to our workshop. Faculty were immersed in five clinical situations, which were depicted through photos, videos or sound recordings of their everyday environment. This successful experiential learning allowed learners to interpret their experiences, give them meaning and plan new actions following a simulated situation based on the reality of their daily settings [17]. Using a mobile audience response tool, faculty were able to express opinions and make selections during various stages of the process, including selecting the EPA, giving feedback to the mock resident and evaluating the resident's entrustment. All responses were anonymously shared with the entire group in real‐time. Following each case, faculty had the opportunity to discuss their impressions, feedback, misunderstandings and important takeaways with guidance from the two instructors. This approach enhanced their overall experiences and provided many opportunities for reflection.

In addition to the components of Stefan et al. on faculty development, our intervention helped to reinforce a shared mental model around EPAs [23], that is, a common basis for understanding and applying the conditions for EPA assessments. Indeed, the completion of EPAs relies on five entrustment factors [24]: the supervisor (as an experienced physician with legitimacy to evaluate the learner), the learner (as a resident committed and motivated to be observed to develop skills), the supervisor–learner relationship (as a frequent and dynamic interpersonal bond with good alignment of expectations), the context (as a safe and appropriate environment for learning) and the task (as a medical activity with reasonable complexity for the learner's skills). Thanks to our workshop, we had a direct impact on two factors: (1) the supervisors, who gained competence in supervising and assessing EPAs, and (2) the relationship between supervisor and learner, which was clarified by better alignment of expectations for EPA completion.

The results of our intervention remain moderate, however. This can be explained by the fact that we only targeted faculty: Despite the improvement in their skills as described below, some residents showed resistance, which can be explained by the fractured learning environment, that is, a discontinuity in supervision [25]. Indeed, as the residents' schedules were not aligned with those of the supervisors, interactions between supervisors and supervisees were not longitudinal, thus altering the relationship of trust between the two [26, 27, 28]. Parallel construction of schedules could have improved continuity of resident supervision by the same clinicians [24]. Another potential explanation for the observed phenomenon may be found in the overall burden of EPA assessments, particularly in a setting characterised by persistent patient overload, interruptions and a lack of time [26, 27]. Although these contextual factors cannot be fully addressed by a workshop, any faculty development initiative must consider them as they remain significant challenges for the completion of EPA assessments. Dedicating an out‐of‐clinic faculty for EPA supervision could improve this situation [28].

### 4.1 Limitations

Our study has certain limitations. First, it is possible that factors other than participating in the workshop may have contributed to a higher rate of EPA assessment than would be expected. For example, it is possible that faculty who did not participate in the workshop had access to the rotation specific cards or felt motivated by the ‘noise’ created by the workshop. The naturally increasing familiarity with EPAs that occurs over time among the participants is another example. Second, the study was conducted in a single centre, focusing on a single ectopic rotation within EM residency programmes. However, because the majority of EM residents in the province complete their paediatric emergency rotations in our institution, we chose to focus on the training of faculty within our department. A third limitation of our study is that we did not account for patient flow during the study period, which was particularly affected by the Covid‐19 pandemic and changes in health care policy. For instance, the emergency department experienced a significant increase in workload at the beginning of March 2021 following the lifting of restrictions, which may have resulted in fewer opportunities for EPA supervision and assessment. A correlation between the number of patients seen in a day and the number of EPAs documented could potentially address this hypothesis.

## 5 Conclusion

EM residents were able to receive more EPA assessments while on their PEM ectopic rotation due to faculty training that utilises multiple strategies, including relevant content selection, experiential learning and numerous opportunities for feedback and reflection. The findings of our study could guide future research in nonpaediatric EM rotations or any programme facing challenges in obtaining a sufficient number of EPA assessments.

## Author Contributions

**Michael Buyck:** conceptualization, methodology, investigation, writing – original draft, formal analysis, writing – review and editing. **Pierre Desaulniers:** conceptualization, supervision, writing – review and editing. **Christophe Chénier:** conceptualization, methodology, writing – review and editing, formal analysis, supervision. **Ahmed Moussa:** conceptualization, methodology, supervision, writing – review and editing, formal analysis.

## Conflicts of Interest

The authors declare no conflicts of interest.

## Supporting information


**Appendix S1.** Supporting Information.
