# Supplementary material for: Experiential Faculty Development to Increase the Number of Entrustable Professional Activity Assessments
Source: Clin Teach. 2024 Dec 25;22(1):e70006. doi: 10.1111/tct.70006 (PMC11669488; doi:10.1111/tct.70006)
Supplement: Supplementary file 1 — Appendix S1. Supporting Information. [file TCT-22-e70006-s001.docx]

# Appendix

Rotation-specific reference card pediatric Entrustable Professional Activity from Residency Programs in Emergency Medicine


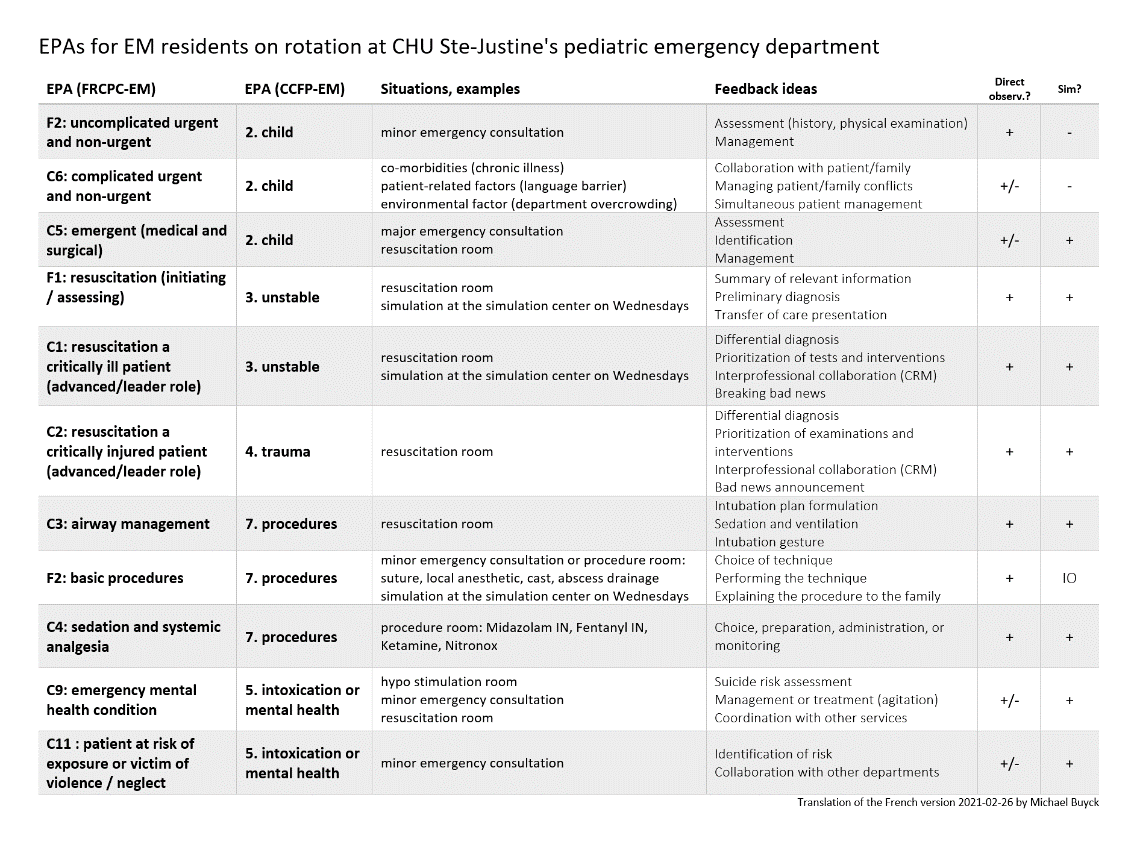

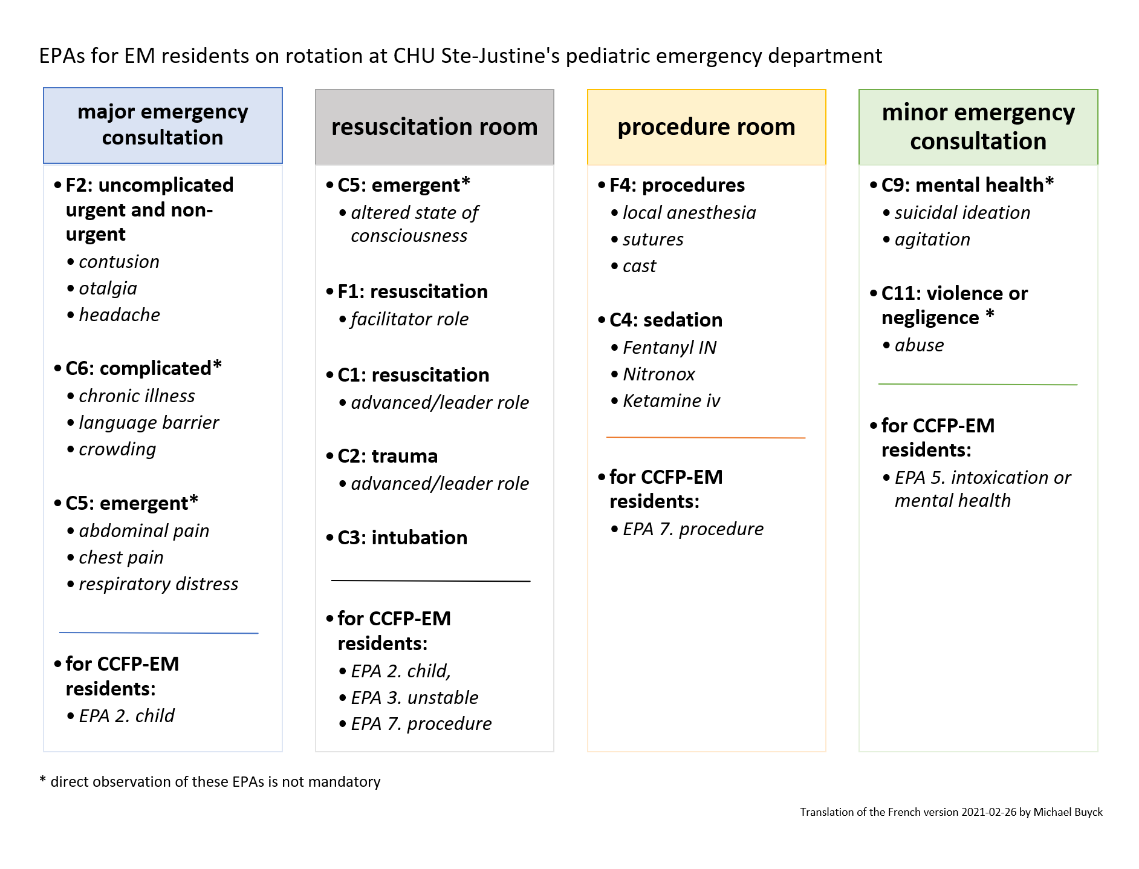


Direct observ.? = Is direct observation mandatory to validate the EPA?

Sim = can the EPA be completed in a simulated situation?

This rotation card is a simplified version of the original French version, which provided environment-specific references (e.g. room numbers, work area designations, etc.). The original is available on request from the authors
